# The Effect of Ultrasonic Treatment on the Binding of the Inclusion Complex β-Cyclodextrin-peppermint Oil with Cellulose Material

**DOI:** 10.3390/ma15020470

**Published:** 2022-01-08

**Authors:** Sandra Flinčec Grgac, Jasna Jablan, Sara Inić, Rajna Malinar, Ivančica Kovaček, Ivana Čorak

**Affiliations:** 1 Department of Textile Chemistry and Ecology, Faculty of Textile Technology, University of Zagreb, HR-10000 Zagreb, Croatia; inicsara15@gmail.com (S.I.); m.rajna@gmail.com (R.M.); ivana.corak@ttf.unizg.hr (I.Č.); 2 Department of Analytical Chemistry, Faculty of Pharmacy and Biochemistry, University of Zagreb, HR-10000 Zagreb, Croatia; jjablan@pharma.hr; 3 Division of Food and Consumer Goods Microbiology, Institute of Public Health “Dr. Andrija Štampar”, HR-10000 Zagreb, Croatia; ivancica.kovacek@stampar.hr

**Keywords:** inclusion complexes β-cyclodextrin-peppermint essential oil, cotton, cotton/PES, 1,2,3,4-butane tetra carboxylic acid, ultrasonic bath, FTIR-ATR, mechanical properties, antimicrobial activity

## Abstract

The purpose of the research was to measure the increase in the binding of inclusion complexes β-cyclodextrin-peppermint oil (β-CD_PM) to cellulose in cotton and cotton/polyester material with BTCA as the crosslinking agent by applying an ultrasonic bath at room temperature and a frequency of 80 kHz for 10 min. After sonication, the samples were left in a bath for 24 h after which they were dried, thermocondensed and subjected to a number of wash cycles. The treated samples were analysed with Attenuated total reflection (ATR) units heated up to 300 °C (Golden Gate (FTIR-ATR)) to monitor chemical changes indicative of crosslinking, while physico-chemical changes in the samples were monitored by using Fourier transform infrared spectroscopy (FTIR-ATR). Mechanical properties were measured according to EN ISO 13934-1:1999, and coloristic changes were evaluated by the whiteness degree according to CIE (WCIE) and the yellowing index (YI), while antimicrobial activity was determined according to AATCC TM 147-2016. The results show a physico-chemical modification of the UZV-treated cellulosic material. Moreover, partial antimicrobial efficacy on Gram-negative bacteria was confirmed for treated fabrics.

## 1. Introduction

Cyclodextrins are cyclic oligosaccharides consisting of (α-1,4)-linked α-d-glucopyranose units with a hydrophilic outer surface and a lipophilic central cavity. Cyclodextrins appear as α-cyclodextrin (α-CD), β-cyclodextrin (β-CD) and γ-cyclodextrin (γ-CD), containing six, seven and eight glucopyranose units, respectively. The central cavity of cyclodextrin is lined by skeletal carbons and ethereal oxygens of glucose residues, which provides it a relatively lipophilic character. On the other side, the hydroxyl groups of glucopyranose units are oriented to the exterior of the molecule, giving it a hydrophilic character [1,2,3]. Due to such structure, cyclodextrins can form inclusion complexes by encapsulating a significant number of molecules of suitable size in their central cavities, either partially or entirely. Cyclodextrins behave as empty capsules (“hosts”) that can incorporate non-polar substances in their lipophilic cavity to form inclusion complexes. Guest molecules can be in a solid, liquid or gaseous state of aggregation depending on complexation conditions. It is known that various molecules from the group of aldehydes, ketones, aliphatic hydrocarbons, amines and various amino acids, fatty acids and aromatic compounds can fit into the cavities of cyclodextrins. It is evident from the above that various active substances such as fragrances, drugs, fungicides or bactericidal agents can be incorporated into the cyclodextrin molecule in such a manner that they form inclusion complexes. The complexing agent is gradually released from cyclodextrin cavities [3,4,5].

Cyclodextrins are very useful and important in the field of medical textiles due to their chemical structure and ability to form inclusion complexes with molecules of other active agents. Due to the high requirements of non-toxicity and moderate antimicrobial activity, many substances are incorporated into β-CD cavities and, as such, applied to the textile substrate. Cyclodextrins are often used to make a complex with essential oils that have healing properties and are environmentally friendly. For many years, peppermint oil, and its ingredients have been used commercially in the food, pharmaceutical and cosmetic industries. Due to its aromatic, antimicrobial and antioxidant properties, it is used as a raw material in toothpastes, mouth fresheners, analgesic balms and for the treatment of the respiratory system. One of the most significant effects of peppermint oil is its inhibitory effect on the growth and development of Gram-positive and Gram-negative microorganisms, and its exceptional antioxidant properties, which are often used to improve human health [6,7].

Numerous authors have studied the possibilities of permanent crosslinking of inclusion complexes β-cyclodextrins with cellulosic material, which occurs when the reactive group of β-cyclodextrins and hydroxyl groups of cellulose interact [3,4,5,8,9,10,11]. For this purpose, researchers worldwide have investigated the possibility of using polycarboxylic acid as a curing agent. There are many studies showing the effectiveness of 1,2,3,4-butane tetra carboxylic acid (BTCA) in binding cyclodextrins to cellulose and polyester fibers. It is known that binding occurs through an esterification reaction between cellulose and/or cyclodextrin by BTCA including two steps where some carboxylic acid groups may remain intact. In the first step, a cyclic anhydride forms between two adjacent carboxylic acid groups. In the second step, the esterification reaction takes place between the previously formed acid anhydrides the hydroxyl groups of the cellulose macromolecules and cyclodextrin to form ester bonds. Insight into research shows that treatment stability has been tested by applying several wash cycles under mild conditions and at lower temperatures. Other scientific research has focused on the modification of CDs with different means to achieve better reactivity with negatively charged substrate surfaces [3,4,5,8,9,10,11,12]. Currently, research covered the possibility of forming inclusion complexes of β-cyclodextrin with peppermint oil using a high-energy vibrating mill as well as the possibility of binding complexes prepared for the textile material of cellulose by ultrasonication using 1,2,3,4-Butanetetracarboxylic acid. The use of ultrasonic treatment was chosen because the effect of ultrasound on textiles causes physical changes in the structure of fibers: they mostly swell, and their absorbency and diffusion coefficient of chemical molecules increases, resulting in better binding of the agent to the material, energy savings, lower water consumption and better processing conditions [13]. The aim of this research was to increase the durability of such treated fabrics during multiple wash cycles at higher temperatures, which, along with the antimicrobial efficacy, is extremely important for their applications in the hospital environment.

## 2. Materials and Methods

In this study, 100% cotton standard fabric WFK 10A DIN 53919/ISO2267 (CO) and a standard polyester/cotton blend (wfk Testgewebe GmbH, Brüggen-Bracht, Germany)in the percentage ratio 65/35 WFK 20 A were used. The properties of both standard fabrics were as follows: mass per unit area 170 g/m^2^; yarn count of warp and weft 27/27 cm^−1^; and linear density 295 dtex, canvas embroidery.

The bath for binding the prepared inclusion complex of β-cyclodextrin (CycloLab R&D Ltd., Budapest, Hungary), and peppermint oil (Sigma Aldrich, St. Louies, MO, USA) (β-CD+PM) to the materials was prepared by dissolving the components in pure water, as shown in Table 1. The pH of the bath thus prepared was 2.46. Due to exceptional acidity, which could completely damage the cellulose material, the pH of the bath was raised to 4.39 by adding 2 mL of 8% NaOH.

The preparation of the inclusion complex of β-cyclodextrin and peppermint essential oil was carried out by mixing β-cyclodextrin with 30% peppermint essential oil based on the mass of β-cyclodextrin. The mixture thus prepared was placed in a high-energy vibrating mill RETSCH^®^-MM 400 (RETSCH^®^, Düsseldorf, Germany) at a frequency of 25 Hz for 10 min and then at a frequency of 10 Hz for 5 min. Subsequently, the mixture was placed in the refrigerator at 4 °C for 24 h.

The samples were treated in an ultrasonic bath at a frequency of 80 kHz, a power of 60 W and a temperature of 25 °C with a sweep program for 10 min.

The treated samples were left in the bath for 24 h. The samples were dried by conduction at 80 °C for 4 min. After drying and before the thermal condensation, the CO_BCD_PM_ H sample was analysed using an FTIR-ATR (PerkinElmer, United States) unit up heated to 300 °C (Golden Gate FTIR-ATR). The treated samples were analysed by an FTIR-ATR Golden Gate unit at temperatures of 150 °C to determine the optimal time for crosslinking the β-CD_PM to the cellulosic material using BTCA as the crosslinking agent. According to the obtained results, the selected time of thermal condensation was 5 min at 150 °C.

After the treatment, some of the samples were subjected to a different number of wash cycles (1, 3 and 10). The washing process was performed in the Wascator FOM71 CLS (Electrolux, Sweden) device according to ISO 6330 6N Textiles Domestic washing and drying procedures for textile testing, with the addition of 20 g of standard detergent. The samples were washed at 60 °C for 66 min. After washing, the samples were dried, ironed and analysed. Table 2 contains the labels and descriptions of the samples. 

All samples were analysed using a spectroscope with the Fourier transform of infrared spectra (FTIR) by applying the attenuated total reflection technique (ATR) (Perkin Elmer, software Spectrum 100, United States). Four scans were performed for each sample at a resolution of 4 cm^−1^ between 4000 cm^−1^ and 380 cm^−1^. For better precision in detecting the links responsible for cross-linking β-cyclodextrin, the samples were treated with 0.1 M NaOH for 4 min prior to measurement and then dried in a drier at 100 °C [13]. The purpose of this treatment was to convert the free carboxyl groups situated in the 1580 cm^−1^ wave number region into carboxylate anions. This left carboxylate esters in the area of 1730 cm^−1^, which were responsible for the bonds of cotton and BTCA, as well as of BTCA and β-cyclodextrin.

In order to evaluate the aesthetic components of the treated fabrics, whiteness and yellowing indexes were measured according to AATCC 110-2000 with lighting D 65/10 on the reflection spectrophotometer Datacolor Spectraflash SF 300 (Lawrenceville, NJ, USA) with the Data Match 300 program.

Tensile properties were measured on the samples in the weft direction before and after the washing cycles according to EN ISO 13934–1:1999 Textiles—Tensile properties of fabrics—Part 1: determination of maximum force and elongation at maximum force using the strip method on a TensoLab Strength Tester (Mesdan S.p.A., Puegnago del Garda, Italy), distance between clamps 100 mm, bursting speed 100 mm/min and pretension 2 N. The mechanical wear as a measure for fabric damage was calculated according to the following:

(1) $$U_{m}=\frac{F_{0}-F}{F_{0}}\times 100\;\left[\%\right]$$

where U_m_ is mechanical damage (wear) (%), *F*_0_ is breaking force of start fabric (N) and *F* is breaking force of treated and/or washed fabric (N). A pair of suitable standard fabrics was used for this calculation. In calculating the mechanical damage to the fabric, in order to establish the influence of the treatment on the material and to exclude the influence of washing, under *F* the breaking force of the treated fabric was taken into account and under *F*_0_ the breaking force of the standard 100% cotton fabric and standard cotton/polyester fabric were taken into account. While calculating the damage after the 3 wash cycles, the breaking force of the standard fabric was washed 3 times (*F*_0_), and the breaking force of the treated fabric washed 3 times (*F*) was taken into account. 

The antimicrobial activity was determined according to AATCC TM 147-2016 Antibacterial Activity Assessment of Textile Materials: Parallel Streak Method.

## 3. Results and Discussion 

**Figure 1:**
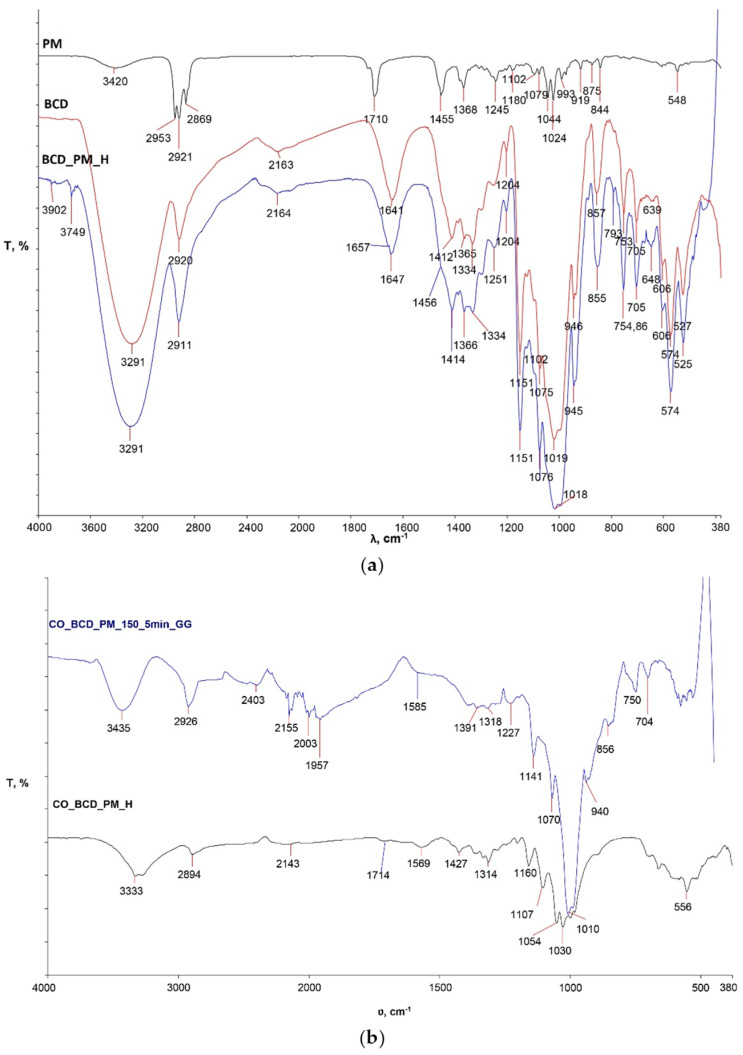
Presentation of physico-chemical changes of the following: (**a**) spectral curves of β-cyclodextrin (BCD) and prepared β-CD inclusion complex with peppermint essential oil (BCD_PM_H); (**b**) the sample CO_BCD_PM_150_5min_GG during analysis on a heated ATR Golden Gate unit at 150 °C for 5 min compared to the FTIR-ATR curve of the treated sample after thermal condensation CO_BCD_PM_H.

Figure 1a shows the spectra of peppermint oil (PM), pure β-cyclodextrin (B-CD) powder used for the study and the inclusion complex of β-cyclodextrin and peppermint oil prepared in the previously described procedure (B-CD_PM_H).

The spectral curve of the inclusion complex B-CD_PM_H coincides with the spectral band of pure β-CD in the entire spectral region. In some parts of the wave number, changes in the intensity of individual peaks are clearly visible, indicating the possible presence of peppermint oil in hydrophobic β-CD cavities. In order to establish the formation of the β-CD_PM inclusion complex in future studies, an analysis of the 1H-NMR values of the chemical shifts will have to be performed.

**Figure 2:**
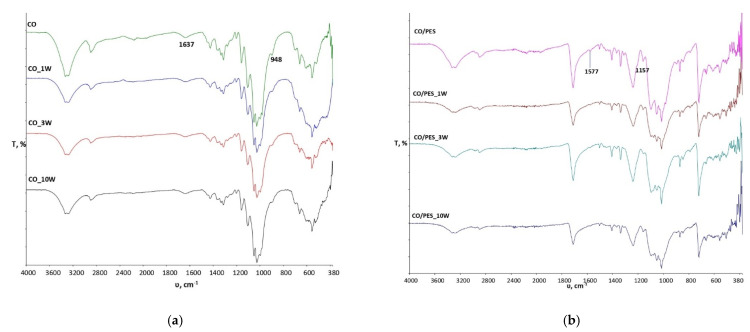
Observation of physico-chemical changes of the following: (**a**) Spectral curves of standard cotton fabric (CO) before and after the 1st, 3rd and 10th washing process (CO_1W, CO_3W and CO_10W). (**b**) Spectral curves of standard cotton fabric (CO/PES) before and after the 1st, 3rd and 10th washing process (CO/PES_1W, CO/PES_3W and CO/PES_10W).

**Figure 3:**
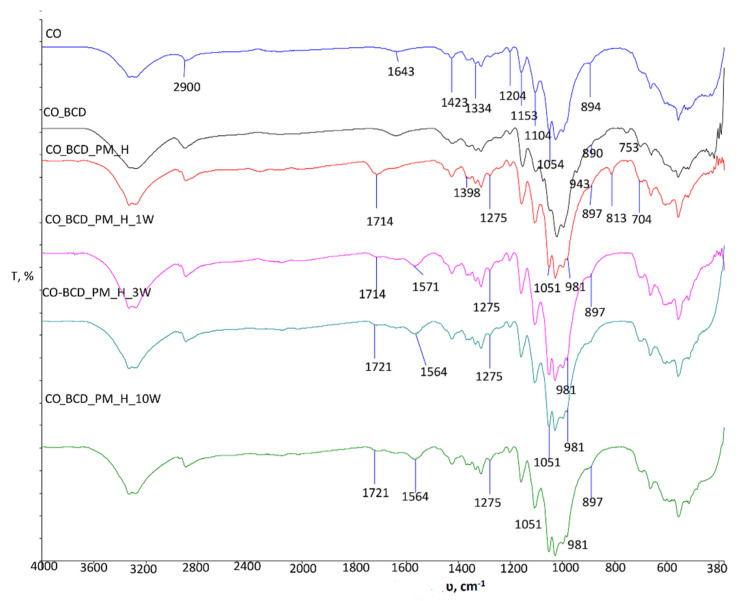
FTIR spectra of the standard cotton and standard cotton samples treated with inclusion complexes of β-cyclodextrin and peppermint oil before and after maintenance cycles.

The high temperature FTIR-ATR Golden Gate unit was used to monitor physico-chemical changes within the treated sample at 150 °C for 5 min to determine the influence of temperature and time on the crosslinking process of the inclusion complex β-CD-PM and cellulose. From the obtained spectral band of the sample CO_BCD_PM_150_5min_GG (Figure 1b), differences in the intensity and wave number of the characteristic peaks are clearly visible in comparison with sample CO_BCD_PM_H, for which its spectral band was recorded with an FTIR-ATR spectrometer. In sample CO_BCD_PM_150_5min_GG, an intense peak at wave number 3435 cm ^−1^ is visible, which is different from the pure cotton material (Figure 2a) and sample CO_BCD_PM_H. Differences are visible on the spectral bands of the cotton sample treated with the inclusion complex of β-CD-PM before and after multiple maintenance cycles (Figure 3), indicating the presence of the inclusion complex β-CD-PM on the surface and in the structure of the treated cotton sample. Peaks on the spectral curve of the treated sample exposed to high temperatures at 1997 and 1969 cm^−1^ indicate bending of the CH bond, i.e., the presence of aromatic groups; a peak at 2349 cm^−1^ indicates the presence of a strong O=C=O bond; and a peak at 2150 cm^−1^ indicates the stretching C=C=O of the bond that is present in ketenes. A peak at 1228 cm^−1^ indicates a strong CO bond, and a peak at 841 cm^−1^ indicates a bending C=C bond of the alkene. In sample CO_BCD_PM_150_5min_GG (Figure 1b), changes are visible in the range from 2403 cm^−1^ to 1957 cm^−1^, which are due to vibrations within aromatic molecules according to many studies (Figure 1b) [14,15].

To gain a better understanding of the changes at the physico-chemical level that occurred during the performance of the 1st, 3rd and 10th washing cycles on standard treated fabrics, the effects of washing on untreated samples of standard fabrics were investigated as well, with the obtained spectra presented in Figure 2. From the spectral bands shown in Figure 2a, it can be observed that the washing process did not cause any significant changes in the physico-chemical properties of the sample of standard cotton fabric even up to 10 cycles. The peak at the wave number 1577 cm^−1^ is caused by vibrations within the C=C group and is visible in the unwashed sample CO/PES, which gradually disappears after multiple wash cycles (Figure 2b).

The differences are also visible at wavenumber 1564 cm^−1^, which is extremely important in the processing of cotton materials in the presence of polycarboxylic acids, as it indicates the formation of free carboxyl groups that are important for the formation of carboxylate esters, whereas the peak in the range of 1700 cm^−1^ to 1740 cm^−1^ indicates the presence of the ester bond, which confirms the binding of the substance (Figure 3). A number of changes in the spectral bands of the treated sample’s are visible in the range of 1398 cm^−1^ to 704 cm^−1^. In the spectral bands of the treated sample CO_BCD_PM_H, the peak appeared at wavenumber 1275 cm^−1^, which is due to the vibration of the saturated ester C-C(=O)-O present in peppermint oil (peak at wavenumber 1245 cm^−1^, Figure 1a). The appearance of peaks with higher intensity at wavenumbers 1100 cm^−1^ and 1054 cm^−1^ after treatment and maintenance cycles is also evidence of the presence of the inclusion complex of β-cyclodextrin-peppermint essential oil on the cellulosic material. The increase in intensity is due to overlapping vibrations within the O-C-O group derived from primary alcohols (1054 cm^−1^) and secondary alcohols (1100 cm^−1^) present in peppermint oil and stretching within the C-O-C in β- glycosidic linkages of cellulose [16,17,18].

**Figure 4:**
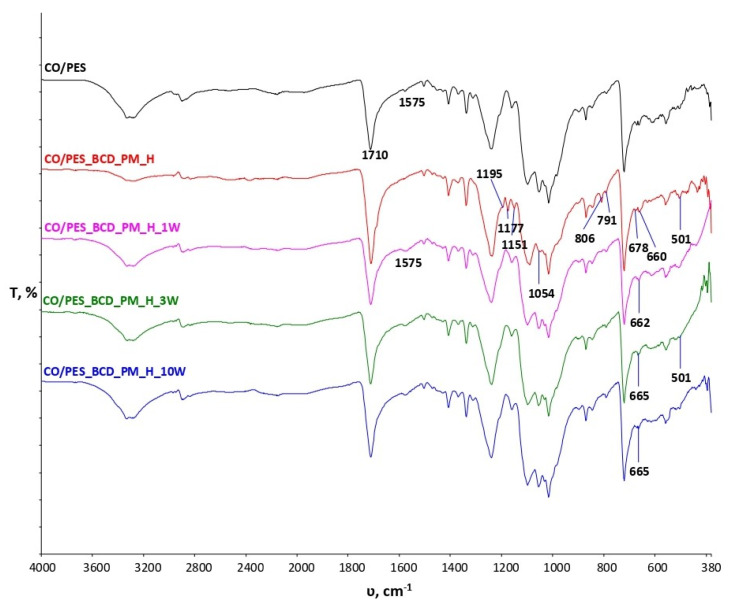
FTIR spectra of standard cotton/polyester (CO/PES) and cotton samples treated with inclusion complexes of β-cyclodextrin and peppermint oil before (CO/PES_B-CD_PM_H) and after maintenance cycles (CO/PES_B-CD_PM_H_1W, CO/PES_B-CD_PM_H_3W, CO/PES_B-CD_PM_H_10W).

Figure 4 shows the spectral curves of cotton/polyester samples treated with inclusion complexes of β-cyclodextrin and peppermint essential oil before and after wash cycles. A peak at 1710 cm^−1^ indicates the presence of an ester group characteristic of polyester fibers, the intensity of which increased significantly after treatment (CO/PES_B-CD_PM_H). The peak at 1575 cm^−1^ is present in the untreated sample with very low intensity. In the treated sample, the peak disappears at this wavenumber, but after one wash cycle the appearance of peaks is visible, confirming the presence of carboxylate anions, indicating the crosslinking of inclusion complexes of β-cyclodextrin peppermint oil with the cellulose component in CO/PES blends by 1, 2, 3, 4-BTCA. The assumption that crosslinking occurred is also confirmed by spectral bands of the untreated CO/PES material after wash cycles in which no peak was present in the scope of 1575 cm^−1^ (Figure 2b). It can also be seen that, in the spectral curve of the treated sample and the treated sample after the 1st, 3rd and 10th wash cycles, there is an increase in peak intensity at 1710 cm^−1^, indicating esterification. However, it is very difficult to determine the degree of esterification due to the presence of ester bonds in the polyester. At wavenumber 1151 cm^−1^ and 1177 cm^−1^, a peak is formed due to stretching within the COCs present in cellulose and β-CD [8,12].

Based on the requirement that the fabric should retain its original properties after processing, which mainly relates to the appearance of the surface, touch, comfort and color, all samples were subjected to a measurement of whiteness and yellowing. It could be observed that the functionalisation of the cotton fabric with the inclusion complex β-CD_PM resulted in a reduction in whiteness, but no significant yellowing occurred. Whiteness and yellowing were evaluated after finishing and the 1st, 3rd and 10th wash cycles; the results are shown in Table 3. 

The change in whiteness or yellowing was observed when the treated fabric was thermocondensed at 150 °C for 5 min. After the first wash cycle, the whiteness of all the samples increased, probably due to the removal of the deposited unbound agent, and after the 3rd and 10th wash cycles the whiteness trend continued and yellowing decreased accordingly. The sample of treated cotton/polyester fabric after 10 wash cycles (CO/ PES_BCD_PM_H_10W) had a slightly lower degree of whiteness (73.0) compared to the untreated sample of standard fabric (CO/PES) (81.6), which indicates a permanent change in the appearance of the material.

It is well known that the thermal condensation process, which is an important part of the durable finishing of cotton, results in the loss of mechanical properties. Measurements were made on untreated and treated fabric before and after the third maintenance cycles in warp and weft directions. In order to determine the effect of finishing on the mechanical properties of the fabric, mechanical damage was calculated from the obtained breaking forces of treated samples (F) (CO_BCD_PM_H, CO/PES_BCD_PM_H) and the breaking forces of standard untreated fabrics (F_0_) cotton and cotton/polyester, (CO, CO /PES). Moreover, to determine the mechanical damage of treated fabrics washed three times, the values of breaking forces of treated fabrics washed three times (F) (CO_BCD_PM_H_3W, CO/PES_BCD_PM_H_3W) and the breaking forces of untreated standard fabrics washed three times (F_0_) (CO0/CO_3W) were taken into account. The obtained results (Table 4) show that the samples of untreated standard cotton fabric have greater mechanical damage after three wash cycles in the warp direction. The same trend is observed for treated cotton standard fabrics with a visible reduction in mechanical damage to fabrics in the warp direction after the third wash cycle. For sample CO_BCD_PM_H_3W, a reduction in mechanical damage in the direction of the warp and weft threads is visible compared to the same unwashed samples (CO_BCD_PM_H). The reason for this is shrinkage during the washing process. This was confirmed by determining the number of threads at 5 cm in warp and weft directions. The number of threads of untreated standard fabric in the warp direction is 28.2 cm^−1^ and 26.6 cm^−1^ in the weft direction. After the third wash cycle, the number of threads of untreated standard fabric increased to 29.6 in warp direction and 29.8 in weft direction. In addition to the shrinkage itself, a possible cause for the reduction in mechanical damage in the sample CO_BCD_PM_H_3W is the removal of unbound substances from the sample surface while reducing the stiffness of the material. 

The samples of the standard fabric CO/PES show greater mechanical damage in the direction of the weft threads after the third wash cycle (CO/PES_3W). The same can be observed in the treated samples before and after the third wash cycle. The samples CO/PES_BCD_PM_H_3W showed a slight increase in mechanical damage compared to treated samples CO/PES_BCD_PM_H.

**Figure 5:**
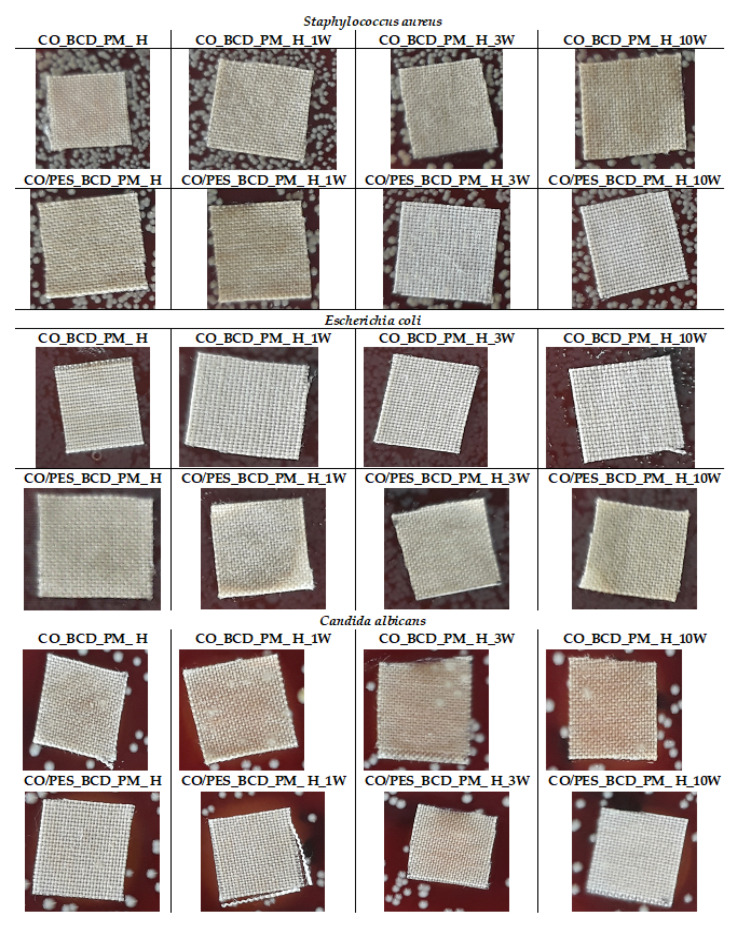
Images of treated and treated washed samples subjected to antimicrobial testing according to AATCC TM 147-2016.

The results of antimicrobial activity for treated samples before and after the maintenance cycles for *Staphylococcus aureus*, *Escherichia coli* and *Candida albicans* are presented in Table 5 and Figure 5. The mechanism of inhibiting the growth and development of the microorganism occurs due to the reaction of phenolic compounds which are active substances in peppermint oil and contain α-pinene, citronellol and methyl eugenol with the cell membrane of the microorganism and, thus, cause its death. The effectiveness of peppermint oil itself in inhibiting the growth of microorganisms depends on the amount and method of application. It has been found that the activity of peppermint oil itself weakens if it is encapsulated or complexed in the carrier as an active substance. Studies of the use of peppermint oil in the food, agro and pharmaceutical industries have shown that its antimicrobial efficacy is lower on *E. coli*. The reason for this lies in the structure of *E. coli* itself, which consists of a double membrane, and the outer membrane has a layer of lipopolysaccharides that prevents the penetration of antibacterial compounds into the cell interior [7].

The results of antimicrobial efficacy on Gram-negative bacteria *Escherichia coli* of the treated cotton sample before (CO_BCD_PM_H) and after the 1st, 3rd and 10th maintenance cycles indicate the absence of an inhibition zone, but bacteria were also not found on and below the sample (Figure 5), which indicates a partial antimicrobial action, we assume the slight release of peppermint essential oil from the inclusion complex. Partial antimicrobial efficacy of the cotton sample after treatment was also demonstrated against Gram-positive *Staphylococcus aureus*. Given the obtained results in further research, the possibility of using higher concentrations of peppermint essential oil in the preparation of an inclusion complex with β-CD in order to achieve better antimicrobial activity will be considered. The sample CO/PES_BCD_PM_H showed partial resistance to *Candida albicans* before and after the 1st, 3rd and 10th maintenance cycle. There are no zones of inhibition, but the effectiveness of the treatment is confirmed by the absence of fungi on the surfaces of the samples (Figure 5). Further research will seek to investigate the effect of the concentration of complex peppermint oil in β_CD cavities on the antimicrobial efficacy of textile substrates treated with such prepared complexes.

## 4. Conclusions

The durable binding of inclusion complexes β-CD_PM to cotton cellulose and its blend with polyester is a challenge for many scientists. In this research, a process of mixing β-CD with peppermint essential oil was first carried out to obtain inclusion complexes. The assumption that inclusion complexes were formed was confirmed by physico-chemical analyses using the FTIR-ATR spectrometer, initially on a sample of β-CD peppermint essential oil inclusive complex (B-CD-PM) and then on all treated samples before and after the wash cycles. The process of finishing standards CO and CO/PES fabrics was performed in an ultrasonic bath with the aim of achieving better bonding of the inclusion complexes β-CD-PM to the cotton and cotton/polyester material. The FTIR-ATR method applied in monitoring physico-chemical properties of the samples clearly indicates spectral band changes and the binding of β-CD_PM inclusion complexes to cellulose by ester bonds, whereas the presence of peaks at 1721 cm^−1^ confirms the persistence of changes after 10 cycles of maintenance. A peak at 1710 cm^−1^ indicates the presence of an ester group characteristic of polyester fibers, with significantly increased intensity after treatment (CO/PES_B-CD_PM_H). The peak at 1575 cm^−1^ is present in the untreated sample, with very low intensity. In the treated sample, the peak disappears at the same wave number, but then after one wash cycle the appearance of peaks is visible, confirming the presence of carboxylate anions, indicating the crosslinking of inclusion complexes of β-cyclodextrin peppermint oil with the cellulose component in CO/PES blends by 1,2,3,4-BTCA. From the obtained results after the 1st, 3rd and 10th wash cycles at 60 °C, it is evident that the binding of β-CD-PM inclusion complexes was successfully performed using ultrasound. Moreover, further research will be focused on the achievement of good mechanical properties using a less acidic medium. The partial antimicrobial efficacy of the CO_BCD-PM_H sample before and after the maintenance cycle can be observed only for *E. coli*. Sample CO/PES_BCD_PM_H showed partial resistance to *Candida albicans* before and after the 1st, 3rd and 10th maintenance cycle. The obtained values indicate the need to increase the amount of peppermint essential oil in future research. 

## Figures and Tables

**Table 1:** The composition of the bath.

Ingredient	Amount
Inclusion complex β-CD+PM	25% by weight of material
1,2,3,4-Butanetetracarboxylic acid (BTCA), 98+%, Alfa Aesar Gmbh&Co	70 g∙L^−1^
Sodium hypophosphite monohydrate (SHP), Sigma Aldrich	65 g∙L^−1^
Felosan RG-N, Bezema	1.5 g∙L^−1^

**Table 2:** Labels and treatment of the cotton (CO) and cotton/PES (CO/PES) fabrics.

Sample	Description of Treatment
CO	Cotton fabric
CO_BCD_PM_ H	Cotton fabric treated with a 1,2,3,4-BTCA solution and inclusion complex β-CD_PM during preparation aged in the refrigerator for 24 h
CO/PES	Cotton/polyester fabric
CO/PES_ BCD_PM_ H	Cotton/polyester fabric treated with a 1,2,3,4-BTCA solution and inclusion complex β-CD_PM during preparation aged in the refrigerator for 24 h
_1W _3W 10W	1st wash cycle 3rd wash cycle 10th wash cycle

**Table 3:** The influence of the finishing and the 1st, 3rd and 10th wash cycle on changes in whiteness (WCIE) and yellowing (YI) standard 100% cotton fabric and CO/PES blends.

Samples	WCIE	YI
CO	73.4	5.48
CO_BCD_PM_H	47.8	14.63
CO_BCD_PM_H_1W	67.3	7.58
CO_BCD_PM_H_3W	77.0	4.32
CO_BCD_PM_H_10W	79.2	3.62
CO/PES	81.6	2.18
CO/PES_ BCD_PM_ H	49.6	13.57
CO/PES_ BCD_PM_ H_1W	69.2	6.51
CO/PES_ BCD_PM_ H_3W	69.8	6.28
CO/PES_ BCD_PM_ H_10W	73.0	5.37

**Table 4:** Average results obtained by testing the strength and elongation of cotton and cotton/polyester specimens before and after treatment and 3 wash cycles.

Samples		F, Breaking Force, N	Ɛ, Elongation, %	Um, Related to Standard Fabric, %
CO	warp	727	8.40	-
	weft	803	21.80	
CO_3W	warp	687	15.10	5.5
	weft	766	24.51	3.36
CO_BCD_PM_H	warp	356	13.50	51.03
	weft	404	19.80	47.26
CO_BCD_PM_H_3W	warp	413	17,20	39.88
	weft	477	20.40	37.73
CO/PES	warp	1044.3	14.90	-
	weft	873	27.90	
CO/PES_3W	warp	1012.0	16.20	3.09
	weft	835.7	27.30	4.27
CO/PES_ BCD_PM_ H	warp	958	17.10	8.26
	weft	732	31.70	16.15
CO/PES_ BCD_PM_ H_3W	warp	925	16.80	8.59
	weft	715	27.00	18.10

**Table 5:** Results of antimicrobial efficacy of samples.

Samples	*Staphylococcus aureus*	*Escherichia coli*	*Candida albicans*
CO	**-**	**-**	**-**
CO_BCD_PM_ H	+/−	+/−	-
CO_BCD_PM_ H_1W	-	+/−	-
CO_BCD_PM_ H_3W	-	+/−	-
CO_BCD_PM_ H_10W	-	+/−	-
CO/PES	-	-	-
CO/PES_ BCD_PM_ H	-	-	+/−
CO/PES_ BCD_PM_ H_1W	-	-	+/−
CO/PES_ BCD_PM_ H_3W	-	-	+/−
CO/PES_ BCD_PM_ H_10W	+/−	-	+/−

+/− partial antimicrobial activity (zone of inhibition cannot be observed, but no colonies beneath); - no antimicrobial activity.

## Data Availability

Not applicable.
